# Information needs of ethnically diverse, vaccine-hesitant parents during decision-making about the HPV vaccine for their adolescent child: a qualitative study

**DOI:** 10.1186/s12889-023-17540-4

**Published:** 2024-01-04

**Authors:** Harriet Fisher, Sarah Denford, Suzanne Audrey, Adam Finn, Huda Hajinur, Matthew Hickman, Sandra Mounier-jack, Asha Mohamed, Marion Roderick, Leanne Tucker, Julie Yates, Tracey Chantler

**Affiliations:** 1 National Institute for Health Research Health Protection Research Unit (NIHR HPRU) in Behavioural Science and Evaluation (BSE), Bristol Medical School, University of Bristol, Bristol, UK; 2 Schools of Population Health Sciences and of Cellular and Molecular Medicine, University of Bristol, Bristol, UK; 3 Caafi Health, Bristol, UK; 4 National Institute for Health Research Health Protection Research Unit (NIHR HPRU) in Vaccinations and Immunisation, London School of Hygiene and Tropical Medicine (LSHTM), Keppel Street, London, UK; 5 LSHTM Vaccine Centre, London, UK; 6 Department of Paediatric Immunology and Infectious Diseases, University Hospitals of Bristol and Weston NHS Foundation Trust, Bristol, UK; 7 UK Health Security Agency, Wellington House, London, UK; 8 Bristol, UK

**Keywords:** HPV vaccine, Qualitative, Vaccine hesitant, Parents, Ethnic minority, Information needs

## Abstract

**Background:**

The English schools-based human papillomavirus (HPV) vaccination programme has the potential to eliminate HPV-related cancers if high uptake is achieved. However, unmet information needs among some parents may contribute to persisting lower uptake among minority ethnic groups. Through this study we aimed to understand the information needs of vaccine-hesitant, ethnically diverse parents during decision-making about the HPV vaccine for their adolescent child, to inform the future development of tailored communication materials.

**Methods:**

Recruitment was facilitated thorough healthcare and community organisations within London and the South West of England. Semi-structured interviews took place between April and August 2023. Thematic analysis was undertaken, assisted by NVivo software.

**Results:**

Of the 29 parents interviewed, the majority were mothers (79%), belonged to a minority ethnic group (88%), and had an adolescent child unvaccinated against HPV (72%). Five of the interviews were undertaken in the participants’ primary language with translation support. Most parents interviewed had limited knowledge about the HPV vaccine and appeared conflicted as to whether vaccines could offer benefits to health. Misunderstanding around the potential of developing serious side-effects (e.g. fertility issues, developing cancer) were factors that could negatively impact decision-making by parents. Stigma associated with the sexual transmissibility of HPV did not always negatively impact decision-making. However, some parents chose not to vaccinate on the basis of perceptions of low risk and a preference to provide education about sexual behaviours to their adolescent child.

**Conclusions:**

Tailoring communication materials to address misunderstandings could support informed decision-making by vaccine hesitant parents for their adolescent children to be vaccinated against HPV. Future communication materials about the HPV vaccine should highlight the benefits of protection against cancer to increase parents’ motivation for protect their adolescent child; provide accurate convincing information in relation to the excellent safety profile; and emphasise the importance of providing HPV vaccine at the recommended age, all alongside communicating the universality and commonality of HPV infection.

**Trial registration:**

N/A.

**Supplementary Information:**

The online version contains supplementary material available at 10.1186/s12889-023-17540-4.

## Highlights

Uptake of the English schools-based HPV vaccination programme has decreased in recent years, and is especially low among young people from minority ethnic groups.

Addressing vaccine hesitant parents’ information needs may support positive HPV vaccine decision-making and improve uptake.

Information needs about the HPV vaccine were elicited through interviews with vaccine hesitant, ethnically diverse parents.

Tailoring communication materials to address misunderstandings could support informed decision-making about the HPV vaccine.

Future communication materials should highlight the benefits of the HPV vaccine, provide transparent information about the safety profile and side-effects, and emphasise the universality and commonality of HPV infection.

## Introduction

Infection with Human Papillomavirus (HPV) can occur through skin-to-skin contact, including sexual contact, and can cause a range of conditions affecting both women and men. These include genital warts and cancers affecting the cervix, vulva, vagina, penis, anus, and oral cavity. The three HPV vaccines – bivalent, quadrivalent, and nonavalent – have proven safety profiles and are efficacious through induction of strong and durable immune responses when administered in early adolescence [1, 2].

In England, the universal HPV vaccination programme is usually offered to adolescent children aged 12–13 years and provided in the school setting. Prior to the Covid-19 pandemic, uptake of the HPV vaccination programme exceeded the 80% threshold required for economic cost-effectiveness [3]. However, inequalities in uptake were shown with young people from minority ethnic groups less likely to receive the HPV vaccine [4, 5]. The most recent data for the 2021/22 HPV vaccination programme shows coverage has fallen to below 70% [6].

Vaccine hesitancy refers to a delay in acceptance or refusal of vaccines despite availability of vaccination services. Vaccine hesitancy is complex and context specific, varying across time, place, and vaccines [5] and is recognised as an important contributor to inequalities in uptake of vaccination programmes [7, 8].

The UK Health Security Agency have produced HPV vaccine information leaflets which are available in 33 different languages, Within the English HPV vaccination programme, these information leaflets and forms requesting parental consent are distributed to parents either electronically or using paper-based forms on behalf of the immunisation team ahead of the scheduled vaccination session. Families from minority ethnic groups are less likely to respond [4]. Opportunities are currently limited within this universal approach for immunisation teams to address vaccine hesitancy or to frame and target specific HPV vaccine messages to parents with additional information needs [7, 8].

Previously with young people from socio-economically deprived and ethnically diverse populations, we jointly co-developed a lesson aiming to address young people’s information needs about the HPV vaccine [11] [available from: https://pshe-association.org.uk/resource/educate-hpv-vaccine]. We are co-designing a multi-component intervention to increase parental confidence in, and adolescents’ access to, the HPV vaccination programme [9]. As part of the current study, we report the information needs of vaccine-hesitant, ethnically diverse parents for decision-making about the HPV vaccine for their adolescent child. The findings will inform the development of new communication materials for such parents.

## Methods

### Study setting

The research was undertaken in areas of London and South West of England, where uptake rates of the HPV vaccination programme are ranked below the national average of 69.6% of the cohort receiving the first dose (61.8% and 68.5%, respectively) [6].

### Recruitment and consent to participate

The National Health Service (NHS) South West Research Ethics Committee and Health Research Authority approved the study (reference: 22/SW/0003).

Initially, immunisation teams were approached to invite parents who had either refused consent, or had not responded to an invitation, to access the 2021/22 schools-based HPV vaccination programme for their adolescent children. Information about the study was provided verbally (by telephone) to parents during a vaccination session or by posting study information to their home address. There were nine responses (including declines). Five of these parents were subsequently interviewed.

Due to the limited recruitment using this approach, community organisations were approached to support the study. Of 33 contacted, two agreed to promote the study. Methods used included an advert within an electronic newsletter, face-to-face contact, and study promotion via social media groups (e.g. WhatsApp). This resulted in recruitment of 24 more parents who were recruited and subsequently interviewed. Community recruitment was not restricted to parents who were invited to the HPV vaccination programme during the 2021/22 programme year. Some participants had multiple teenage children eligible for the HPV vaccine.

Topic guides were designed to capture parents’ perspectives on: (i) childhood vaccines; (ii) HPV vaccine; (iii) HPV vaccine information for parents; (iv) the parental consent process; and (v) consent and decision-making (see Supplementary File 1).

Interviews were undertaken by the study researchers (HF, TC, SD) and took place either by telephone or digital platform according to participant preference. Interviews were in English, or in the participants’ preferred language facilitated by a translator with appropriate language skills organised by Caafi Health Community Interest Company (https://www.caafihealth.org.uk/). Interviews lasted on average 42 min (range: 22–75 min).

Written or verbal consent was obtained from all participants before the semi-structured interviews commenced. Whenever specific consent was offered, interviews were digitally recorded. Two participants refused recording, but agreed for written notes to be taken which were included as part of the dataset.

Recordings were transcribed verbatim and thematic analysis [10] was undertaken assisted by NVivo 12 software. Both inductive and deductive approaches were employed to analyse the content, focusing on the main research question while identifying key information needs emerging from the data. Coding of all transcripts was undertaken by one researcher (HF), while a second researcher (TC) double-coded a sub-set of six transcripts to check for meaning, relevance and reliability, and to agree the coding framework applied to the full set of transcripts. Themes were identified within which similarities and differences represented within the data were explored. An analysis meeting was also held with members of the wider research team (HF, TC, LT, SD) to discuss the relevance of emerging themes.

In presenting the results, participants have been numbered to protect anonymity, with descriptors to indicate the ethnicity by which they described themselves, the gender of their adolescent child, and their vaccination status at the time of the interview (Table 1).

**Table 1:** Socio-demographic characteristics of interview participants

**Interview**	**Gender**	**Location**	**Ethnicity**	Gender of vaccine eligible teenager	**Vaccination status of child**	**Time of interview (minutes)**	
1	Female	Bristol	White British	Male	Unvaccinated	50	
2	Male	Bristol	British Asian	Male and female	Unvaccinated	36	
3	Male	London (Hackney)	Black British	Male and female	Vaccinated	41	
4	Male	London (Hackney)	Black British	Male	Unvaccinated	37	
5	Female	Bristol	White British	Female	Unvaccinated	65	
6	Male	London (Hackney)	Black Caribbean	Male	Unvaccinated	41	
7	Male	London (Hackney)	British South Asian	Female	Unvaccinated	57	
8	Male	London (Hackney)	Black American	Male and female	Vaccinated	44	
9	Female	London (Islington)	Black	Male	Unvaccinated	52	
10	Female	Bristol	White British	Male	Unvaccinated	51	
11	Female	Bristol	Somali	Male	Unvaccinated	32	
12	Female	London (Tower Hamlets)	Somali	Male	Unvaccinated	46	
13	Female	London (Tower Hamlets)	Bengali	Male	Unvaccinated	42	
14	Female	London (Tower Hamlets)	British African	Male	Vaccinated	22	
15	Female	Bristol	Somali	Female	Unvaccinated	49	
16	Female	Bristol	African	Male	Vaccinated	41	
17	Female	Birmingham	Somalian	Female	Unvaccinated	Interview not recorded	
18	Female	Bristol	British African	Male and female	Vaccinated	23	
19	Female	Bristol	Bengali	Male	Unvaccinated	36	
20	Female	Bristol	British African	Not provided	Unvaccinated	42	
21	Female	Bristol	Sudanese	Female	Vaccinated	50	
22	Female	Bristol	Somali	Not provided	Unvaccinated	47	
23	Female	London	Black African	Female	Unvaccinated	23	
24	Female	Bristol	Somali	Male and female	Unvaccinated	41	
25	Female	Bristol	White British	Male and female	Unvaccinated	25	
26	Female	Bristol	Somali	Not provided	Vaccinated	40	
27	Female	London	Somali	Not provided	Vaccinated	Interview not recorded	
28	Female	Bristol	African Somali	Female	Unvaccinated	45	
29	Female	Bristol	Somali	Not provided	unvaccinated	37	

## Results

The overarching themes presented below related to the following themes and corresponding sub-themes related to the information needs influencing decision-making of ethnically diverse, vaccine hesitant parents: protection (‘levels of awareness and knowledge’, ‘perceptions of benefit’, and ‘information needs’), perceptions of harm (‘safety and side-effects’ and ‘accessibility of information’), and sexual transmission (‘risk perceptions’ and ‘knowledge gaps’). Illustrative quotations were chosen which express concisely, and typify, responses relating to the themes. Where participants were not speaking in their first language, or with translation support, their words are presented verbatim or as those of the translator.

### Theme: Protection

#### Levels of awareness and knowledge

The majority of parents had little knowledge about the benefits and risks of their adolescent children receiving the HPV vaccine, with many parents confirming that this was influential to their adolescent child not receiving the HPV vaccine:

*‘[My son] has been offered by the school and I said no because 1) I didn’t have knowledge and 2) I wasn’t too sure what’s the vaccination for and why is it necessary for me to give my child the vaccine.*’ [Participant 12, mother of unvaccinated adolescent boys, Somali];

‘*All my kids had a vaccine as babies. If they tried to educate me on [the HPV vaccine], then obviously I would have said yes*.’ [Participant 13, mother of unvaccinated adolescent boys, Bengali].

If communication about the HPV vaccination programme was only provided in English, this further contributed to a lack of understanding. It was also acknowledged to be influential within the wider community:

‘*Where I live the language barriers are quite high. I think if it [HPV vaccine information] came home in different languages it would be helpful*.’ [Participant 14, mother of vaccinated adolescent boys, British African];

‘*There are mums and dads who don’t speak English. If you print in their own language and get a platform where they communicate in different tongues and say it is okay and this is what it is*.’ [Participant 20, mother of unvaccinated adolescent, British African].

#### Perceptions of benefit

Often, parents appeared to be uncertain about the balance of risk and benefit of the vaccine. While most interview participants opted for their children to receive vaccines offered during early infancy, many reported that they refused, or delayed, vaccinations offered beyond 12 months:

‘*[Interview participant] has vaccinated her younger children already and given them childhood vaccinations, she hasn’t given him his school booster vaccine. She said in general she’s 50/50 when it comes to vaccination*.’ [Participant 28, mother of unvaccinated adolescent child, African Somali, verbatim quote from translator].

There appeared to be hesitancy to vaccinate among a few parents because of a perception of adolescents being healthy and therefore not in need of protection:

‘*Those extra immunisations, teenage years, are not really something that they need – it’s optional*.’ [Participant 15, mother of unvaccinated adolescent girl, Somali];

‘*They’re teenagers, they’re strong, and so what are they protected against, I don’t understand*.’ [Participant 24, mother of unvaccinated adolescent boys and girls, Somali].

The recent expansion of the HPV vaccination programme to include adolescent boys, appeared to add further factors contributing to a more cautious approach during decision-making for their adolescent sons. Parents were unclear as to whether there would be health benefits from the HPV vaccine for boys:

‘*I’m not an anti-vaxxer or anything, but it was more oh, I didn’t know the boys could have it. How long has this been around for? Is it proven benefits? Why would I or why wouldn’t I do this?*’ [Participant 01, mother of unvaccinated adolescent boy, White British];

‘*My first thought would have been [boys are vaccinated] to protect females… In terms of long term effects I don’t know what the effects are on males not having had the injection*.’ [Participant 25, mother of unvaccinated adolescent boy and girl, White British].

*Information needs* There was variation in the extent to which HPV vaccine information provided to parents appeared to address parents’ information needs. Some parents felt the information was sufficient to inform their decision-making. However, other parents felt the information was insufficient and reported undertaking their own research to address their information needs.

‘*I remember having to look it up [information about the HPV vaccine]. There wasn’t any information in it which I read which gave me any idea of the data*.’ [Participant 2, father of unvaccinated teenage boys and girl, British Asian].

Despite having vaccine-eligible adolescent children, some parents reported not receiving any information about the HPV vaccination programme.

‘*I wasn’t given any information or I was never told about the HPV vaccination process by the school*.’ [Participant 7, father of unvaccinated teenage girl, British South Asian].

Many of the participants of this study were receptive to hearing information about the HPV vaccine or asked questions about the HPV during the interview. Some parents suggested more information was required, in relation to evidence of disease burden and the effectiveness of the HPV vaccine in reducing associated illnesses, to support decision-making:

‘*What are the differences between statistics back before the HPV vaccine and statistics now? What’s the difference between if you do have it and if you don’t?*’ [Participant 05, mother of unvaccinated adolescent daughter, White British];

‘*For what reason have they developed this, is it because it’s more apparent because people are being diagnosed with it, or that particular cancer?*’ [Participant 19, mother of unvaccinated adolescent boys and girls, Bengali].

### Theme: perception of harm

#### Safety and side-effects

The potential for harm from the HPV vaccine was an important factor in decision-making among parents. There was acknowledgement from parents that, as with all medicines, there would be the potential for side-effects. Minor side-effects that would last for a short period did not appear to be a major cause for concern or reason not to vaccinate:

‘*Every medicine has got side effects. I think you have to explain the side effects. You have to explain the benefits and you have to make sure they combat that fear*.’ [Participant 20, mother of unvaccinated adolescent girl, British African];

‘*If there’s only aches and stuff, that’s fine, as long as nothing major that it’s going to have a long-time condition or something*.’ [Participant 13, mother of unvaccinated adolescent boys, Bengali].

However, many parents discussed major concerns and anxieties around the potential for serious side-effects from the HPV vaccine. Often parents raised fears about the potential for serious, long-term harm, although they did not describe specific health conditions that they were concerned might be attributable to the HPV vaccine:

‘*I think I came across once a programme or a video where some young girls had serious side effects from having the vaccine and it led to some sort of health complications*.’ [Participant 15, mother of unvaccinated adolescent girl, Somali];

‘*I wasn’t sure how my son’s body was going to react to it – if my son’s body did react to it, what can they do? What else could be done? To stop that negative reaction. Is it going to have more destruction to his body than actually beneficial*.’ [Participant 16, mother of vaccinated adolescent boys, African].

Specific side-effects mentioned included potential impact on fertility, causing cancer, and developmental issues:

‘*Her [interview participant] main concern is that she’s heard that the side effects of the HPV vaccine could cause cancer and it could cause infertility*.’ [Participant 28, mother of unvaccinated adolescent girl, African Somali, verbatim quote from translator];

‘*Everyone they’re thinking any child, any vaccine is causing huge problem. Children with autistic is rising dramatically in the last five to eight years.*’ [Participant 12, mother of unvaccinated adolescent boys, Somali].

#### Accessible information

A perceived lack of accessible information about side-effects contributed to distrust among some parents and influenced decision-making:

‘*What I was more concerned about is – and I think it’s a common thing in any sort of initiative or something that the NHS does – they [healthcare professionals] never mention where people had side effects*.’ [Participant 15, mother of unvaccinated adolescent girl, Somali];

‘*Most vaccinations you’re not given all the information with what’s wrong, are you?*’ [Participant 18, mother of vaccinated adolescent boys and girls, British African];

‘*I think it’s [vaccine information] all just like one sided so I tend to kind of read other things and other people’s perspective and other studies and see if it’s safe*.’ [Participant 23, mother of unvaccinated adolescent girl, Black African].

A few parents felt reassured about vaccinating their adolescent child because they had spoken to friends or family members whose adolescent children had received vaccine:

‘*I was concerned more about the side effects. Everyone I‘ve spoken to their child hasn’t been affected or anything so that was fine*.’ [Participant 14, mother of vaccinated adolescent boys, British African].

### Theme: sexual transmission

#### Perceptions of risk

Respondents varied in the extent that perceptions of sexual transmission were influential in decision-making. It was difficult for some parents to acknowledge that their child could be sexually active, and therefore be at risk of acquiring HPV. This related both to cultural norms and also perceptions of their child’s development/emotional maturity:

‘*Coming from Islamic orthodox background we don’t condone or promote sexual promiscuity at all, or associate with it, in any forms*.’ [Participant 11, mother of unvaccinated adolescent boys, Somali;

‘*It wasn’t like she was going out and at risk of sexually assault and it wasn’t like she was choosing to have a relationship, she wasn’t partying, she wasn’t getting drunk. She wasn’t in any of those social situations*.’ [Participant 25, mother of unvaccinated adolescent son and daughter, White British].

However, other parents were able to balance their own expectations of sexual behaviours with perceptions of generational changes around sexual behaviours within their community:

‘*My mum, she’d go, ‘Oh, we won’t need that then, no. My child won’t need that. It’ll be fine.’ But no, definitely I’m aware of what’s out there and how easy it is to pick up something from someone*.’ [Participant 11, mother of unvaccinated adolescent boys, Somali].

In some cases, moral overtones were evident where some parents emphasised a preference to provide education to their adolescent child to prevent them engaging in ‘risky’ sexual behaviours:

‘*My wife was saying there’s an education layer here as well for [adolescent daughter] around [the HPV vaccine] which I think I’m pretty on top of, she is a wild child but she’s not stupid as well*.’ [Participant 02, father of unvaccinated adolescent son and daughter, British Asian];

‘*They’re [adolescent children] not supposed to have a sexual intercourse without getting married. So that bit I wasn’t worried about it because I know we work hard to teach our kids and educate, but some of them go in the wrong way and there’s no control over it*.’ [Participant 12, mother of unvaccinated adolescent boys, Somali].

Open discussions about the need for the HPV vaccine and decision-making, both with the wider community and within their family, could be problematic because of perceptions of sex being a stigmatised subject:

‘*In the South Asian community there’s a lot of taboo attached to the concept around sex in general and HPV. If it’s a vaccination around sex, then there’s a lot of misconception and judgement*.’ [Participant 07, father of unvaccinated adolescent daughter, British South Asian];

‘*I like to be honest with my kids and discuss anything even though it’s difficult especially within my culture, it’s not something that we discuss*.’ [Participant 24, mother of unvaccinated adolescent boys and girls, Somali].

#### Knowledge gaps

While most parents were aware that the HPV vaccine offers protection against a sexually transmitted infection, there was sometimes a lack of understanding about how sexual activity could lead to the development of cancer:

‘*How it is related to the sexual [contact] and the cancer, I wasn’t too sure. I was a bit confused*.’ [Participant 12, mother of unvaccinated adolescent boys, Somali];

‘*She [interview participant] thought it [development of HPV-related cancer] was more the development of some type of cells within the human body and this vaccination prevent that cancer from happening, but she never understood it comes from an infection itself*.’ [Participant 21, mother of vaccinated adolescent girl, Sudanese, verbatim quote from translator].

Where parents perceived that their adolescent child was at a lower risk of acquiring HPV this often related to the age at which it is routinely offered (12 − 13 years) and the misconception that the vaccine was provided in anticipation of sexual contact:

‘*I’m not really comfortable about the idea of giving him the vaccine because I don’t see any need why he should be given it, because I know he might not be prone to any sexually transmitted disease*.’ [Participant 06, father of unvaccinated adolescent son, Black Caribbean].

In these cases, parents preferred for the HPV vaccine to be delayed until their adolescent child was older or more likely to be sexually active:

‘*I feel like children are encouraged to be sexual and 12 years old is basically too young to deal with those stuff. Maybe like if they were 15 or upwards it’s understandable*.’ [Participant 14, mother of adolescent vaccinated boys, British African].

Other parents supported the current age the HPV vaccine was offered to ensure protection ahead of sexual debut:

‘*I just think it [HPV] would be something that he [adolescent son] may come across when he gets a bit older – I could use it as a reason to actually take it*.’ [Participant 14, mother of vaccinated adolescent boys, British African];

‘*My understanding is by doing the HPV vaccination now, first dose, presumably the logic is that by the time they do the second dose they’re then protected in time for them being sexually active*.’ [Participant 01, mother of unvaccinated adolescent son, White British].

## Discussion

This study has examined parental information needs during decision-making about the HPV vaccine for their adolescent children, from the perspective of vaccine hesitant, ethnically diverse parents. Anxieties related to the potential for serious, long-term side-effects (e.g. fertility issues, developmental delays) appeared to negatively overshadow the protection offered against developing HPV-related cancers. Stigma associated with protection against a sexually transmitted infection did not always negatively influence parental decision-making, but misunderstandings about the importance of vaccination during early adolescence could influence parents to delay vaccination of their adolescent children. Some of the misunderstandings highlighted as part of this study may be amenable to change through improved communication about the HPV vaccine. While these concerns have been highlighted elsewhere in the literature, the findings will be used to design a culturally appropriate and acceptable communication intervention for the target audience.

Below, we consider the implications of the study findings on the development of tailored communication materials (e.g. films) that are being produced as part of a wider study [9]. The films will be made available to use alongside, and complement, the existing information leaflets produced by UK Health Security Agency. These findings of this study also have wider implications for the development of other communication materials or strategies to promote the HPV vaccine among vaccine hesitant populations.

### Improving parental knowledge and understanding

Underpinning the decision-making process among many of the parents interviewed was limited knowledge and misunderstandings about HPV and the HPV vaccine. This confirms the findings of previous studies which highlight information needs about the HPV vaccine among parents, including those whose adolescent children have been vaccinated [11–14].

Content of future communication materials should emphasise the clinical sequelae of HPV, protection offered against HPV-related diseases, universality of health benefits by gender, and the effectiveness of the HPV vaccine and vaccination programme. Highlighting the potential benefit of prevention of HPV-related cancer by inclusion of the story of a cancer survivor may help increase parents’ motivation to vaccinate their adolescent children. Future communication materials should be developed in different languages to ensure that parents who do not speak English has their first language are not excluded from the decision-making process. This could also include multi-lingual follow-up letters to ensure that parents who are unable to consent due to lack of awareness, rather than vaccine hesitancy.

### Overcoming parental attitudes to future adolescent sexual activity

The complex influence of parental attitudes towards possible future sexual activity of their adolescent children has been reported widely elsewhere [12–15]. Framing HPV vaccine messages to focus on cancer prevention, appealing to parents’ responsibility to protect their adolescent’s health, rather than sexual transmission could help to address barriers to uptake related to stigma [16]. Perceptions that information about vaccines was withheld from the general public were voiced by participants in this study, alongside requests for open, transparent information about the HPV vaccine. On this basis, we propose that future communication materials, and related HPV messages, should directly address the misconceptions that parents voiced around being able to keep their adolescent children safe from the potential effects of HPV by educating them not to have sexual relations outside of marriage.

A key information need relates to the rationale and need for vaccination during early adolescence, with some parents delaying vaccination until they felt their adolescent child would be engaging in sexual relationships. Messaging should emphasise that the HPV vaccine will provide adolescents with protection for when they do become sexually active, rather than accelerating their sexual debut, and that the vaccine is most effective if provided ahead of potential exposure to HPV. Future communication materials should also promote the universality and commonality of HPV, emphasising that transmission can still occur even in sexual relationships within the context of marriage. Normalisation of vaccination within different communities could be achieved by ensuring film participants (healthcare professionals and parents) are representative of these communities.

### Reassurance for parents

Corroborating findings reported elsewhere [11, 12, 14, 15], potential for harm to their adolescent children and anticipated regret were instrumental during decision-making among the parents interviewed as part of this study. Distrust around the safety of vaccines was apparent, with parents referring to misinformation circulating on social media and perceptions that key information about side-effects was not explicitly provided within official circulated information. As widely trusted sources of vaccine information [17], healthcare professionals could play an important role in dispelling misconceptions and providing reassurance to parents. Parents may be unwilling to raise concerns or not know that vaccinations are approaching. Healthcare professionals could help reassure parents by making reference to upcoming vaccinations at routine checkups.

### Strengths and limitations

This study addresses the limited evidence base within the context of the English HPV vaccination programme in relation to decision-making and information needs about the HPV vaccine among parents belonging to minority ethnic groups [18, 19]. Participants in this study were from a range of ethnic backgrounds with the majority of participants identifying themselves as Black British or Black African as recruitment was predominantly supported through a community organisation with strong links to these groups. We were also able to include key groups whose voices are often excluded from research, including parents who do not speak English as their first language and who are less likely to engage with healthcare services. Although participants from different minority ethnic groups were represented, the findings may not be representative of other minority communities with low vaccine uptake who were not included (e.g. Gypsy and Traveller, Eastern European, Jewish Orthodox). Data on social class was not collected so it was not possible to identify differences in responses by socio-economic background.

## Conclusions

Decision-making among vaccine-hesitant, ethnically diverse parents can be negatively influenced by an overall lack of knowledge, as well as understanding about the diseases and associated harms that the HPV vaccine protects against, anxieties relating to the false perception that there are serious side-effects, and their perceptions of their adolescent child’s future exposure. Future communication materials about the HPV vaccine should highlight the benefits of protection against cancer to increase parents’ motivation for protect their adolescent child; provide accurate convincing information in relation to the excellent safety profile; and emphasise the importance of providing HPV vaccine at the recommended age, all alongside communicating the universality and commonality of HPV infection.

### Electronic supplementary material

Below is the link to the electronic supplementary material.

Supplementary Material 1: Parent topic guide

## Data Availability

The datasets generated and/or analysed during the current study are not publicly available as the study has not yet been completed [at the time of manuscript submission] but are available from the corresponding author on reasonable request.

## Abbreviations

HPV: Human papillomavirus
NHS: National Health Service
