# Supplementary material for: Information needs of ethnically diverse, vaccine-hesitant parents during decision-making about the HPV vaccine for their adolescent child: a qualitative study
Source: BMC Public Health. 2024 Jan 4;24:91. doi: 10.1186/s12889-023-17540-4 (PMC10768213; doi:10.1186/s12889-023-17540-4)
Supplement: Supplementary file 1 — Supplementary Material 1: Parent topic guide [file 12889_2023_17540_MOESM1_ESM.docx]

**Parent Topic Guide**

**Explain purpose of project and session**

We are trying to find out from parents what concerns they may have about the HPV vaccine. We would also like to design an intervention which can be used to help parents make informed decisions about the HPV vaccine and to make it easier to get the vaccine if they want their teenager to have it. We will use what people tell us in these interviews to create a preliminary plan for an intervention to help improve delivery of the HPV vaccination programme. We anticipate that the interview will last up to one hour.

Today, we would like to ask for your views on the HPV vaccine, existing communication materials (e.g. videos, leaflets), and views on how an intervention could be designed. We are also talking to professionals involved in delivery of the HPV vaccination programme

[Note: *Ensure that the participant has read the information sheet and signed the consent form or provided verbal consent before the interview starts*.]

**Childhood vaccines**

Did your child have the recommended vaccines during childhood?

If no, what were the reasons for this?

**HPV vaccine**

Before your teenager was offered the HPV vaccine, had you ever heard of HPV?

Can you tell me what you know now about HPV/HPV vaccine?

What do you think the positive things about the HPV vaccine are?

What do you think the negative things about the HPV vaccine are?

**Information for parents**

How did you find out about the vaccine?

What information were you given and by whom? What did you think about this information (trust, answer their questions, easy to understand)?

Who did you talk to about the HPV vaccine?

What other information did you look for?

**The parental consent process**

How did you find out your teenager was going to be offered the HPV vaccination?

Did you discuss it with your teenager? If yes, what did you say? Prompt: Did you think it was a good/bad idea for them to have the vaccination?

Were there any disagreements about the HPV vaccine? E.g. one of you thinking it was a good idea to have the vaccine and another thinking it wasn’t? If yes, how was the disagreement sorted out?

Did you receive a form or email?

Did you complete this?

Did you receive any reminders to send the form back to the school? If yes, how? Prompt: email, telephone, through your teenager?

Our records show that your parent/carer form was not returned to the school. Do you know why?

Do you think sending out a form to parents/carers is a good way of getting consent for the HPV vaccine? Can you think of any other ways? Prompt: Email? Text? Opt-out? Consent once for all vaccines? Young people consent?

**Consent and decision making**

Did your teenager receive the HPV vaccine? *[Note: Expected that vaccine not received]*

Who decided whether your teenager should have/not have the vaccine?

Can you tell me a little bit about how this decision was made?

What were the most important reasons why your teenager did not have the vaccine?

- Trust in public health programmes
- Government intrusion in private lives

**Review of existing communication materials**

*Show participant example of communication material and allow participant to read the information or watch the video.*

- ‘[Vaccines_ Myths and facts - Caring for Kids](file:///C:\Users\epxsa\AppData\Local\Study%20data\Study%20One\Parent%20vaccination%20materials\Canada\Vaccines_%20Myths%20and%20facts%20-%20Caring%20for%20Kids.pdf)’ leaflet
- ‘[paul-roebuck-my-mouth-cancer-story](file:///C:\\Users\\epxsa\\AppData\\Local\\Study%20data\\Study%20One\\Parent%20vaccination%20materials\\Oral%20Health%20Foundation\\paul-roebuck-my-mouth-cancer-story.mp4)’ video, Oral Health foundation
- ‘All about HPV’ animation video, EDUCATE

- ‘[surveillance-of-side-effects-of-the-hpv-vaccine](file:///C:\Users\epxsa\AppData\Local\Study%20data\Study%20One\Parent%20vaccination%20materials\WHO\surveillance-of-side-effects-of-the-hpv-vaccine.mp4)’ video, WHO

*Ask the participant to provide feedback on the different communication materials.*

- What did you like about the leaflet / video?
- What did you not like about it?
- What are the most important messages from the leaflet / video?
- How relevant does the leaflet / video feel to you? (e.g. relatability of participants, ethnicity)
- Is there anything you would prefer to see more of?
- What is the most important thing you would change?
- Do you think the content of the video / leaflet is presented clearly or not?
- Were you able to understand all of the content?
- Were any of the content or words used difficult to understand?
- Could any of the messages / content be worded differently?
- Is there any additional content that you think is important to include?

**Developing communication materials**

*As part of this study, we are hoping to design new communication materials to complement the existing leaflet produced for the English HPV vaccination programme. [Show participant HPV vaccine leaflet]*

What format do you think the new materials should be?

- Videos
- Leaflets
- Web pages

What information do you think are the most important things to include?

- How HPV is spread
- Diseases that HPV can cause
- Need for vaccination (e.g. not sexually active, cultural and religious beliefs)
- Target age when vaccination offered
- Safety
- Side-effects
- Research into vaccine
- Anything else?

* What do you think are the three most important things that should be included?

If we produce videos, what scenarios do you think we could include?

- Interview with healthcare professionals
- Interview with cancer survivor
- Parents talking about making decisions about the HPV vaccine
- Parents and children talking about HPV vaccination
- Animation about vaccine safety data monitoring and safety

* What do you think are the three most important things that should be included?

**Strategies to improve communication about the HPV vaccine**

*We are particularly interested in reaching parents who did not provide consent for their teenager to be vaccinated.*

Do you think parents who did not provide consent would be willing to discuss vaccination with any of the following:

- Faith leaders
- Social workers, community workers
- Health visitors
- General practice staff
- Pharmacists
- Other parents whose children have had the vaccine
- Teenagers
- School staff
- Immunisation nurses

* What do you think are the three most important things that should be included?

How could we share information with parents (best ways to reach them)?

- Media campaigns
- Parents evening at school
- Health fairs / events
- Community advocates / vaccine champions
- General practices
- One-to-one with healthcare professionals
- Web-based forums
- Interactive information sessions

* What do you think are the three most important things that should be included?

**Strategies to improve access to the HPV vaccine**

*Ask participant to recommend strategies most likely to improve access where parents do not provide consent for the HPV vaccination to be given at the scheduled school-based session but subsequently want their child to be vaccinated*.

- Additional catch-up session in school
- Recall by general practice
- Availability of the vaccine at pharmacies / community organisations.
- Allowing young person to consent themselves

**Improving uptake of childhood vaccinations**

Do you think we should be doing more to make sure children receive the recommended vaccines?

Whose responsibility do you think it is to make sure children are vaccinated?

- GP
- Family responsibility
- Other healthcare professionals
- Social workers

What could we do to make it easier for families to get the vaccines?

- Family clinics
- Incentives
- Pharmacy led clinics
- Social media campaigns
- Catch up session in schools

**Finally, is there anything else you would like to tell me or ask me about?**

**Many thanks for taking part in this interview.**

[NOTE: *ensure that the participant is given the £30 gift voucher and reimburse travel expenses*]
